# Supplementary figures and images for: Differences by sex and type of hypertension in mortality from hypertensive diseases between 1997 and 2020, and predictions for 2035 in Latin American and Caribbean countries
Source: PLoS One. 2026 Mar 2;21(3):e0342267. doi: 10.1371/journal.pone.0342267 (PMC12952635; doi:10.1371/journal.pone.0342267)

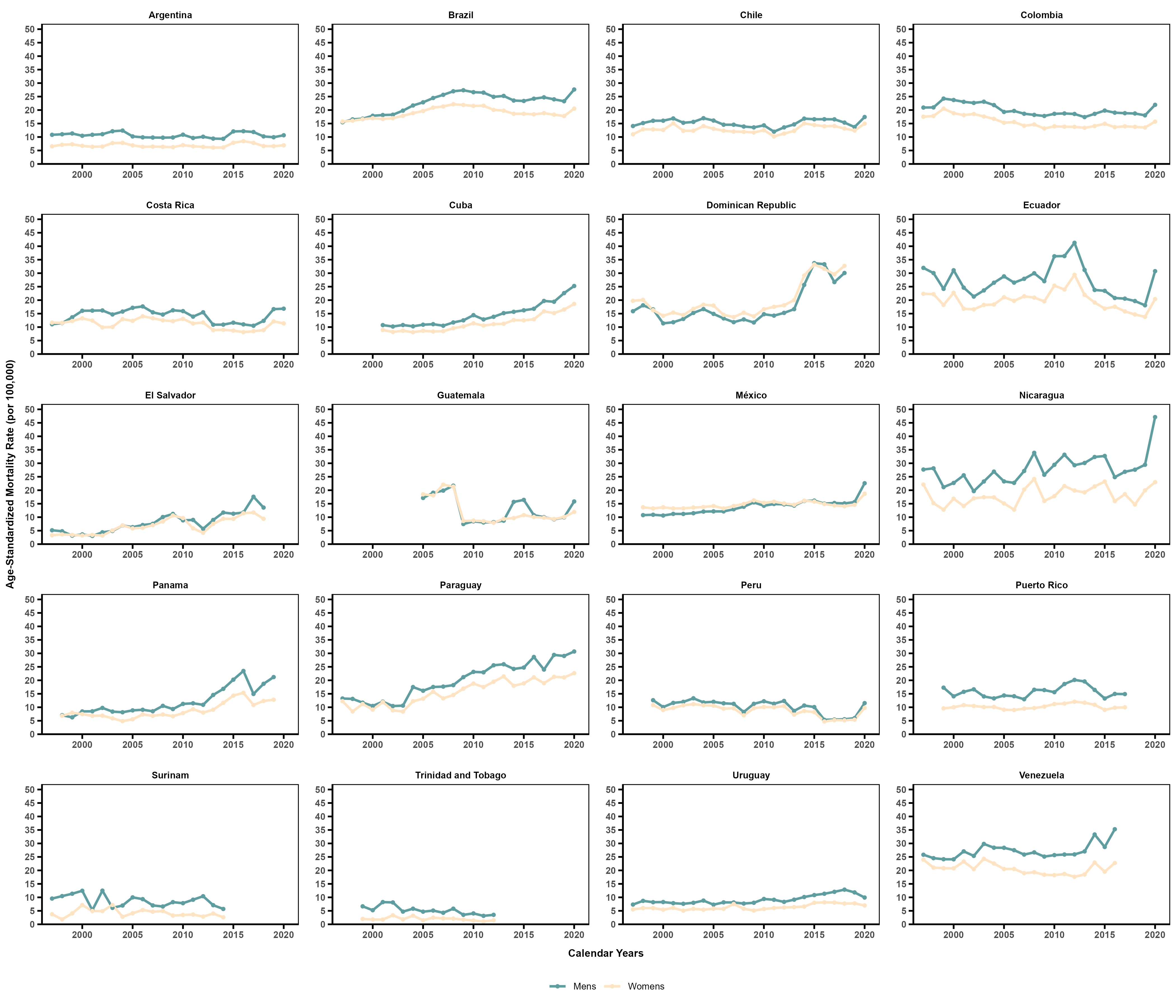

Supplement: S1 Fig — (TIFF) [file pone.0342267.s001.tiff]

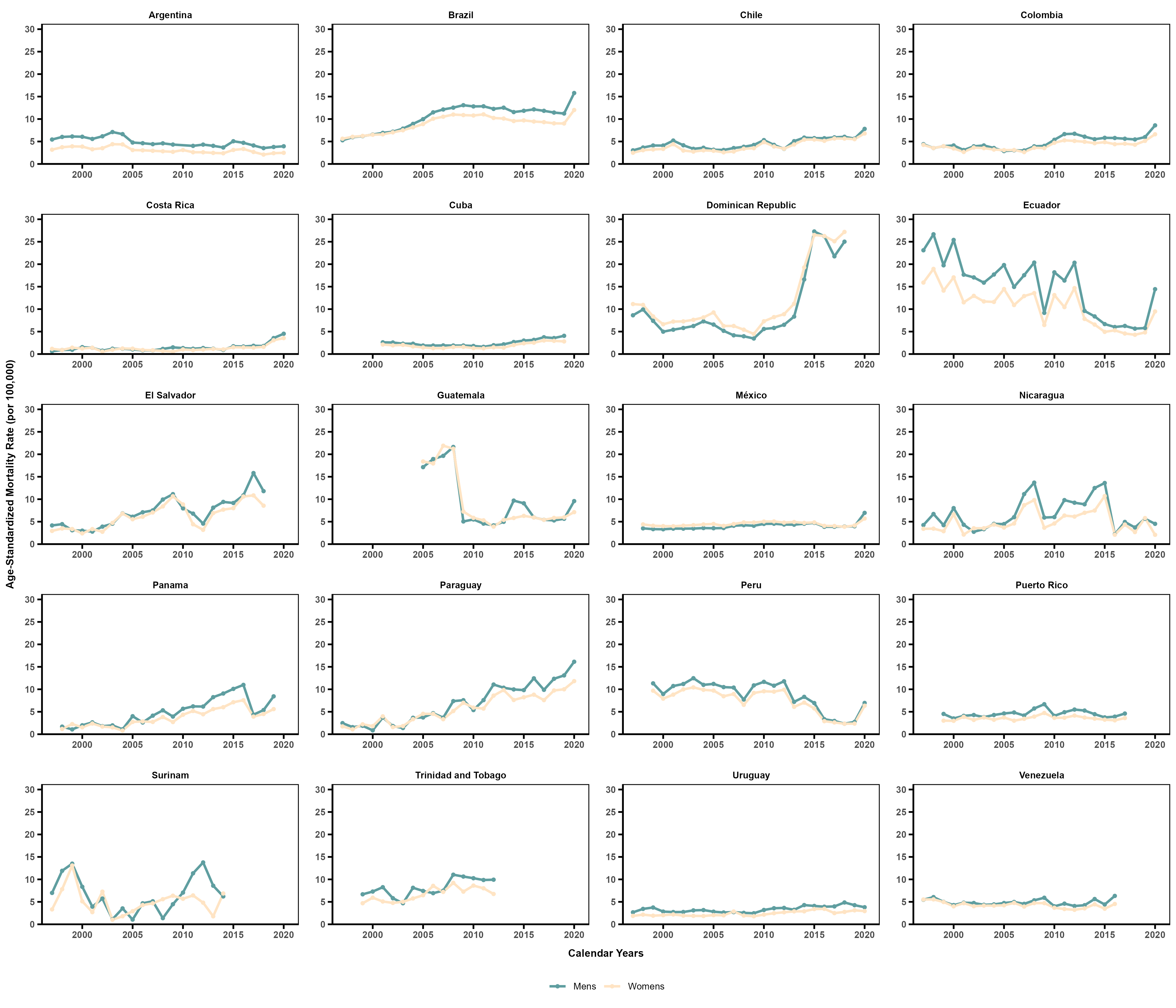

Supplement: S2 Fig — (TIFF) [file pone.0342267.s002.tiff]

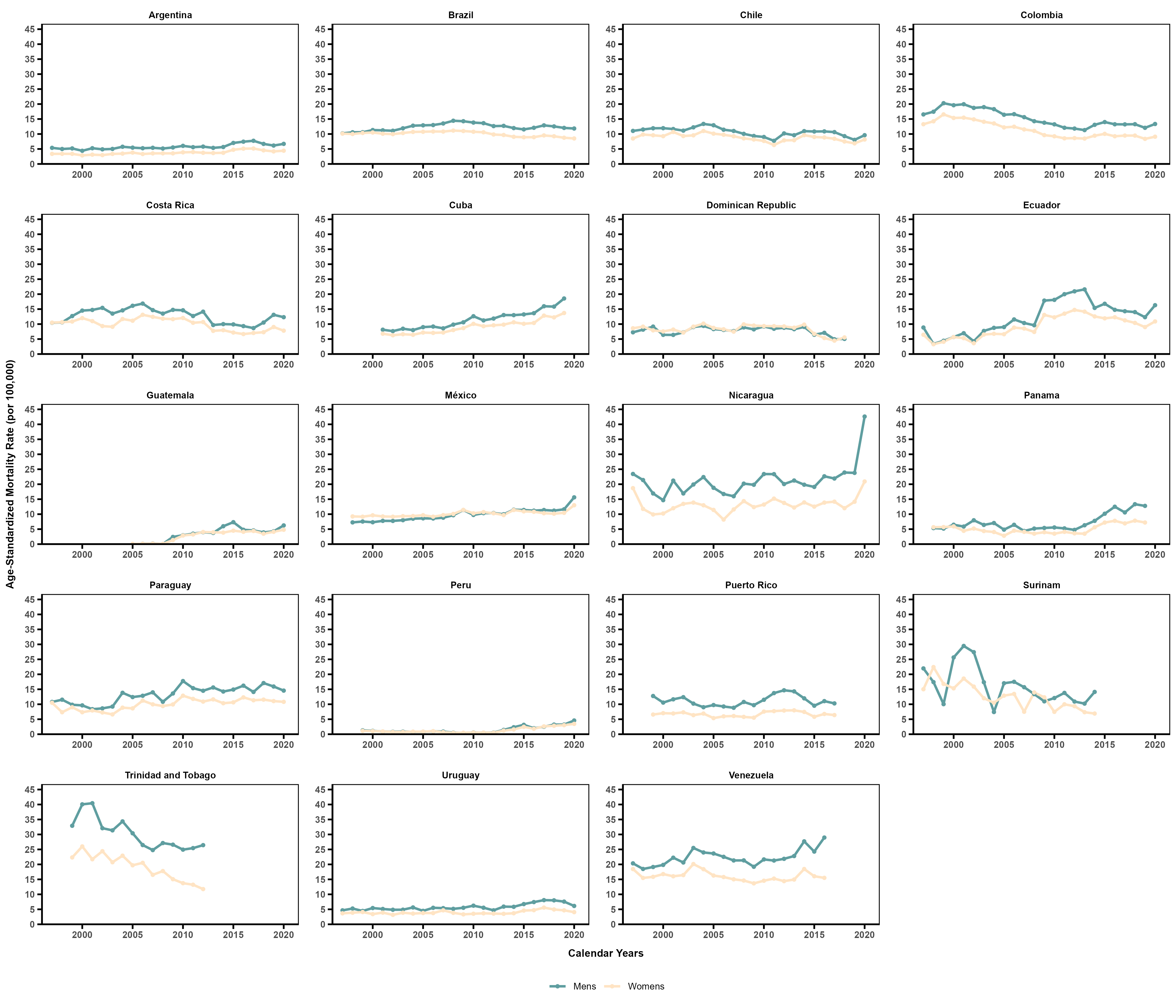

Supplement: S3 Fig — (TIFF) [file pone.0342267.s003.tiff]
