# Supplementary material for: Differences by sex and type of hypertension in mortality from hypertensive diseases between 1997 and 2020, and predictions for 2035 in Latin American and Caribbean countries
Source: PLoS One. 2026 Mar 2;21(3):e0342267. doi: 10.1371/journal.pone.0342267 (PMC12952635; doi:10.1371/journal.pone.0342267)
Supplement: S1 Table — (DOCX) [file pone.0342267.s004.docx]

**S1 Table. Annual percentage change in mortality trends from hypertensive diseases (I10-I13) for men in twenty countries in Latin America and the Caribbean, 1997 to 2020.**

| **Countries** | **Years** | **APC** | **Years** | **APC** | **Years** | **APC** | **AAPC** |
| --- | --- | --- | --- | --- | --- | --- | --- |
| Argentina | 1997−2020 | −0.1(−0.7,0.4) |  |  |  |  | −0.1(−0.7,0.4) |
| Brasil | 1997−2008 | 5.5*(4.7,6.3) | 2008-2018 | −1.4*(−2.2,−0.7) | 2018-2020 | 7.3(−0.5,15.8) | 2.6*(1.8,3.4) |
| Chile | 1997−2011 | −1.7*(−2.9,−0.4) | 2011-2014 | 7.1(−13.3,32.3) | 2014-2020 | 0.6(−3.5,2.4) | −0.3(−3.0,2.5) |
| Colombia | 1997−2000 | 5.5(−4.6,16.7) | 2000-2009 | −3.5*(−5.4,−1.5) | 2009-2020 | 1.1*(0.1,2.1) | −0.2(−1.7,1.3) |
| Costa Rica | 1997−2006 | 3.9*(0.6,7.3) | 2006-2017 | −4.6*(−6.7,−2.4) | 2017-2020 | 19.2*(5.7,34.5) | 1.6(−0.6,3.7) |
| Cuba | 2001−2015 | 3.6*(2.7,4.5) | 2015-2020 | 9.5*(6.3,12.8) |  |  | 5.1*(4.1,6.1) |
| Dominican Republic | 1997−2012 | −0.8(−2.6,1.0) | 2012-2015 | 36.0(−1.3,87.6) | 2015-2018 | −5.4(−16.2,6.7) | 3.0(−1.6,7.9) |
| Ecuador | 1997−2012 | 2.3*(0.6,4.0) | 2012-2018 | −10.9*(−17.6,−3.7) | 2018-2020 | 29.8(−7.0,81.2) | 0.7(−2.7,4.2) |
| El Salvador | 1997−2018 | 6.6*(4.8,8.4) |  |  |  |  | 6.6*(4.8,8.4) |
| Guatemala | 2005−2020 | −2.9(−6.5,0.7) |  |  |  |  | −2.9(−6.5,0.7) |
| México | 1998−2015 | 2.6*(2.1, 3.1) | 2015-2018 | −4.0(−13.8,6.9) | 2018-2020 | 23.1*(12.1, 35.2) | 3.4*(1.7, 5.1) |
| Nicaragua | 1997−2018 | 0.7(−0.2,1.8) | 2018-2020 | 23.1(−2.3,55.1) |  |  | 2.5*(0.4, 4.7) |
| Panama | 1998−2019 | 6.1*(4.7, 7.5) |  |  |  |  | 6.1*(4.7, 7.5) |
| Paraguay | 1997−2012 | 5.8*(4.1, 7.4) | 2012-2020 | 2.6*(0.1, 5.1) |  |  | 4.6*(3.3, 6.0) |
| Peru | 1999−2014 | −0.8(−2.5,0.9) | 2014-2018 | −19.0(−34.8,0.4) | 2018-2020 | 54.9*(3.3, 132.1) | −0.4(−5.5,4.9) |
| Puerto Rico | 1999−2007 | −2.0(−5.2,1.2) | 2007-2012 | 7.5(−1.1,17.0) | 2012-2017 | −7.0*(−12.2,−1.5) | −0.9(−3.7,1.9) |
| Surinam | 1997−2014 | −2.0(−4.2,0.2) |  |  |  |  | −2.0(−4.2,0.2) |
| Trinidad and Tobago | 1999−2012 | −5.7*(−8.3,−3.0) |  |  |  |  | −5.7*(−8.3,−3.0) |
| Uruguay | 1997−2012 | 0.6(−0.1,1.4) | 2012-2018 | 7.2*(3.4,11.1) | 2018-2020 | −12.1(−24.4,2.3) | 1.1(−0.4,2.7) |
| Venezuela | 1997−2012 | 0.1(−0.9,1.1) | 2012-2016 | 6.5*(0.4,13.0) |  |  | 1.4*(0.1,2.8) |

***: p < 0.05 indicates statistical significance. APC: Annual Percent Change; AAPC: Average Annual Percent Change.**
