# Supplementary material for: Differences by sex and type of hypertension in mortality from hypertensive diseases between 1997 and 2020, and predictions for 2035 in Latin American and Caribbean countries
Source: PLoS One. 2026 Mar 2;21(3):e0342267. doi: 10.1371/journal.pone.0342267 (PMC12952635; doi:10.1371/journal.pone.0342267)
Supplement: S2 Table — (DOCX) [file pone.0342267.s005.docx]

**S2 Table. Annual percentage change in mortality trends from hypertensive diseases (I10-I13) for women in twenty countries in Latin America and the Caribbean, 1997 to 2020.**

| **Countries** | **Years** | **APC** | **Years** | **APC** | **Years** | **APC** | **AAPC** |
| --- | --- | --- | --- | --- | --- | --- | --- |
| Argentina | 1997−2020 | 0.2 (−0.4,0.8) |  |  |  |  | 0.2(−0.4,0.8) |
| Brasil | 1997−2008 | 3.5*(2.8, 4.2) | 2008-2018 | −2.1*(−2.8,−1.5) | 2018-2020 | 6.1(−0.9,13.5) | 1.2*(0.5,1.9) |
| Chile | 1997−2020 | 0.5(−0.1,1.1) |  |  |  |  | 0.5(−0.1,1.1) |
| Colombia | 1997−1999 | 8.3(−8.3,27.9) | 1999-2009 | −3.9*(−5.2,−2.5) | 2009-2020 | 0.6(−0.2,1.5) | 0.7(−2.2,0.8) |
| Costa Rica | 1997−2010 | 0.7(−0.9,2.4) | 2010-2016 | −8.0*(−13.5,−2.2) | 2016-2020 | 11.4*(3.1,20.4) | 0.1(−2.0,2.3) |
| Cuba | 2001−2006 | −0.8(−6.0,4.7) | 2006-2020 | 5.5*(4.6,6.3) |  |  | 3.8*(2.3,5.3) |
| Dominican Republic | 1997−2010 | −1.6(−3.5,0.2) | 2010-2015 | 17.0*(7.2, 27.7) | 2015-2018 | −0.3(−10.3,10.6) | 2.6*(0.1, 5.3) |
| Ecuador | 1997−2012 | 1.9*(0.5,3.3) | 2012-2018 | −9.0*(−14.6,−3.1) | 2018-2020 | 18.3(−10.2,55.7) | 0.2(−2.6,3.1) |
| El Salvador | 1997−2009 | 11.4*(7.7,15.3) | 2009-2012 | −12.5(−41.2,30.2) | 2012-2018 |  | 7.6*(1.6,13.9) |
| Guatemala | 2005−2007 | 16.6(−8.3,48.3) | 2007-2010 | −29.2*(−46.2,−7.0) | 2010-2020 | 2.7*(0.6,4.9) | −3.0(−8.2,2.5) |
| México | 1998−2015 | 1.1*(0.6, 1.6) | 2015-2018 | −5.2(−14.8,5.3) | 2018-2020 | 16.0*(5.4, 27.6) | 1.5(−0.1,3.1) |
| Nicaragua | 1997−2020 | 0.9(−0.1,1.9) |  |  |  |  | 0.9(−0.1,1.9) |
| Panama | 1998−2012 | 1.3(−1.1,3.8) | 2012-2015 | 22.5(−14.8,76.4) | 2015-2019 | −4.1(−12.4,4.9) | 3.0(−2.2,8.5) |
| Paraguay | 1997−2020 | 3.7*(2.9, 4.6) |  |  |  |  | 3.7*(2.9, 4.6) |
| Peru | 1999−2012 | −0.3(−2.6,1.8) | 2012-2018 | −11.3*(−19.6,−2.3) | 2018-2020 | 40.2(−5.1,107.4) | −0.4(−4.8,4.0) |
| Puerto Rico | 1999−2007 | −1.2(−3.5,1.0) | 2007-2012 | 5.0(−1.1,11.5) | 2012-2017 | −4.6*(−8.2,−0.9) | −0.5(−2.5,1.4) |
| Surinam | 1997−2014 | −2.9(−5.8,0.1) |  |  |  |  | −2.9(−5.8,0.1) |
| Trinidad and Tobago | 1999−2012 | −3.9(−8.1,0.4) |  |  |  |  | −3.9(−8.1,0.4) |
| Uruguay | 1997−2012 | 0.2(−0.8,1.4) | 2012-2016 | 8.6(−3.3,22.0) | 2016-2020 | −3.4(−9.9,3.6) | 1.0(−1.2,3.3) |
| Venezuela | 1997−2012 | −1.6*(−2.6,−0.6) | 2012-2016 | 5.5(−0.6,12.1) |  |  | −0.1(−1.5,1.2) |

***: p < 0.05 indicates statistical significance. APC: Annual Percent Change; AAPC: Average Annual Percent Change.**
