# Supplementary material for: Differences by sex and type of hypertension in mortality from hypertensive diseases between 1997 and 2020, and predictions for 2035 in Latin American and Caribbean countries
Source: PLoS One. 2026 Mar 2;21(3):e0342267. doi: 10.1371/journal.pone.0342267 (PMC12952635; doi:10.1371/journal.pone.0342267)
Supplement: S3 Table — (DOCX) [file pone.0342267.s006.docx]

**S3 Table. Average annual percent change and 95% confidence intervals for primary hypertension (I10) for men in twenty countries in Latin America and the Caribbean, 1997 to 2020.**

| **Countries** | **Years** | **APC** | **Years** | **APC** | **Years** | **APC** | **Years** | **APC** | **AAPC** |
| --- | --- | --- | --- | --- | --- | --- | --- | --- | --- |
| Argentina | 1997-2020 | −2.2*(−3.0,−1.5) |  |  |  |  |  |  | −2.2*(−3.0,−1.5) |
| Brasil | 1997-2008 | 9.0*(8.0,10.0) | 2008-2018 | −1.4*(−2.2,−0.5) | 2018-2020 | 15.2*(6.3,24.7) |  |  | 4.8*(3.9,5.7) |
| Chile | 1997-2020 | 3.2*(2.1,4.3) |  |  |  |  |  |  | 3.2*(2.1,4.3) |
| Colombia | 1997-2007 | −3.2*(−5.8,−0.5) | 2007-2011 | 21.1(6.4,37.8) | 2011-2018 | −3.0*(−6.1,0.1) | 2018-2020 | 25.4*(6.9,47.1) | 2.9*(0.1,5.8) |
| Costa Rica | 1997-2017 | 2.5*(0.5,4.6) | 2017-2020 | 43.9*(20.4,72.1) |  |  |  |  | 7.2*(4.3,10.1) |
| Cuba | 2001-2011 | −4.2*(−5.7,−2.6) | 2011-2015 | 16.4*(5.1,−27.7) | 2015-2019 | 7.3*(2.6,12.1) |  |  | 2.5*(0.3,4.8) |
| Dominican Republic | 1997-2011 | −4.5*(−7.4,−1.6) | 2011-2015 | 57.1*(26.1,95.8) | 2015-2018 | −3.1(−14.8,10.1) |  |  | −22.2*(−31.6,−11.5) |
| Ecuador | 1997-2012 | −2.5*(−4.6,−0.4) | 2012-2018 | −19.2*(−30.2,−6.6) | 2018-2020 | 79.3*(0.8,218.9) |  |  | −2.1(−7.8,3.8) |
| El Salvador | 1997-2009 | 11.2*(6.6,16.1) | 2009-2012 | −15.0*(−49.1,41.9) | 2012-2018 | 15.8*(7.5,24.4) |  |  | 8.2*(0.6,16.5) |
| Guatemala | 2005-2008 | 2.0*(−26.3,41.3) | 2008-2011 | −38.9*(−79.9,86.3) | 2011-2020 | 7.0*(−1.7,16.6) |  |  | −5.2*(−22.5,16.0) |
| México | 1998-2015 | 2.1*(1.6,2.6) | 2015-2018 | −9.2(−19.1,1.7) | 2018-2020 | 37.6*(24.9,51.6) |  |  | 3.2*(1.5,5.0) |
| Nicaragua | 1997-2014 | 5.5*(0.6,10.7) | 2014-2020 | −16.5*(−28.7,−2.2) |  |  |  |  | −0.7(−5.6,4.4) |
| Panama | 1998-2015 | 12.3*(8.0,16.8) | 2015-2019 | −9.4(−22.9,6.4) |  |  |  |  | 7.8*(3.4,12.3) |
| Paraguay | 1997-2020 | 9.1*(7.3,10.9) |  |  |  |  |  |  | 9.1*(7.3,10.9) |
| Peru | 1999-2012 | 0.4(−1.9,2.9) | 2012-2018 | −22.2*(−31.6,−11.5) | 2018-2020 | −61.9(−11.5,196.5) |  |  | −2.2(−8.3,4.1) |
| Puerto Rico | 1999-2017 | 0.4(−1.1,2.0) |  |  |  |  |  |  | 0.4(−1.1,2.0) |
| Surinam | 1997-2004 | −18.8(−37.5,5.4) | 2004-2014 | 13.7*(0.2,29..0) |  |  |  |  | −1.0(−12.1,11.4) |
| Trinidad and Tobago | 1999-2012 | 4.0*(1.6,6.5) |  |  |  |  |  |  | 4.0*(1.6,6.5) |
| Uruguay | 1997-2007 | −1.3(−4.3,1.7) | 2007-2020 | 3.9*(2.1,5.8) |  |  |  |  | 1.6*(0.0,3.2) |
| Venezuela | 1997-2016 | 0(−1.0,1.2) |  |  |  |  |  |  | 0(−1.0,1.2) |

***: p < 0.05 indicates statistical significance. APC: Annual Percent Change; AAPC: Average Annual Percent Change.**
