# Supplementary material for: Differences by sex and type of hypertension in mortality from hypertensive diseases between 1997 and 2020, and predictions for 2035 in Latin American and Caribbean countries
Source: PLoS One. 2026 Mar 2;21(3):e0342267. doi: 10.1371/journal.pone.0342267 (PMC12952635; doi:10.1371/journal.pone.0342267)
Supplement: S4 Table — (DOCX) [file pone.0342267.s007.docx]

**S4 Table. Average annual percent change and 95% confidence intervals for primary hypertension (I10) for women in twenty countries in Latin America and the Caribbean, 1997 to 2020.**

| **Countries** | **Years** | **APC** | **Years** | **APC** | **Years** | **APC** | **Years** | **APC** | **AAPC** |
| --- | --- | --- | --- | --- | --- | --- | --- | --- | --- |
| Argentina | 1997-2020 | −2.0*(−2.9,−1.2) |  |  |  |  |  |  | −2.0*(−2.9,−1.2) |
| Brasil | 1997-2008 | 7.0*(6.2,−7.9) | 2008-2018 | −2.2*(−2.9,−1.5) | 2018-2020 | 13.5*(5.8,−21.8) |  |  | 3.4*(2.6,4.2) |
| Chile | 1997-2020 | 3.9*(2.9,5.0) |  |  |  |  |  |  | 3.9*(2.9,5.0) |
| Colombia | 1997-2007 | −3.1*(−5.2,−0.8) | 2007-2011 | 16.2*(3.8,30.1) | 2011-2018 | −2.9*(−5.9,0.0) | 2018-2020 | 23.9*(6.9,43.6) | 2.2*(−0.2,4.7) |
| Costa Rica | 1997-2014 | −1.0*(−3.8,1.8) | 2014-2020 | 23.9*(14.9,33.5) |  |  |  |  | 4.9*(2.1,7.7) |
| Cuba | 2001-2011 | −4.6*(−7.3,−1.9) | 2011-2017 | 15.9*(8.3,24.0) | 2017-2019 | −3.3*(−23.4,22.0) |  |  | 1.8*(−1.4,5.3) |
| Dominican Republic | 1997-2010 | −4.4*(−7.1,−1.6) | 2010-2015 | 35.5*(20.6,52.2) | 2015-2018 | 1.7(−9.2,13.9) |  |  | 4.7*(1.2,8.3) |
| Ecuador | 1997-2012 | −2.4*(−4.4,-0.3) | 2012-2018 | −17.3*(−27.5,−5.7) | 2018-2020 | 61.5*(−4.0,172.0) |  |  | −2.3(−7.5,3.0) |
| El Salvador | 1997-2009 | 12.3*(8.1,16.7) | 2009-2012 | −18.1*(−49.5,32.6) | 2012-2018 | 13.7*(6.4,21.4) |  |  | 7.7*(0.6,15.3) |
| Guatemala | 2005-2008 | 1.8(−10.0,15.2) | 2008-2011 | −39.3*(−59.4,−9.2) | 2011-2020 | 5.0*(1.5,−8.7) |  |  | −6.4(−13.1,−0.7) |
| México | 1998-2013 | 1.6*(1.0,2.2) | 2013-2018 | −5.7*(−9.4,−1.9) | 2018-2020 | 21.1*(8.2,35.5) |  |  | 1.4*(0.1,2.8) |
| Nicaragua | 1997-2020 | 0.8(−2.5,4.3) |  |  |  |  |  |  | 0.8(−2.5,4.3) |
| Panama | 1998-2015 | 10.1*(6.8,13.5) | 2015-2019 | −8.0(−19.9,5.6) |  |  |  |  | 6.4*(2.9,10.1) |
| Paraguay | 1997-2020 | 7.6*(5.9,9.2) |  |  |  |  |  |  | 7.6*(5.9,9.2) |
| Peru | 1999-2012 | 0.1(−2.1,2.5) | 2012-2018 | −21.5*(−30.5,−11.3) | 2018-2020 | 63.6(−6.7,187.1) |  |  | −2.0(−7.7,3.9) |
| Puerto Rico | 1999-2017 | 0.3(−0.8,1.4) |  |  |  |  |  |  | 0.3(−0.8,1.4) |
| Surinam | 1997-2014 | −1.8(−6.2,2.7) |  |  |  |  |  |  | −1.8(−6.2,2.7) |
| Trinidad and Tobago | 1999-2012 | 4.0*(1.5,6.6) |  |  |  |  |  |  | 4.0*(1.5,6.6) |
| Uruguay | 1997-2020 | 2.4*(1.6,3.3) |  |  |  |  |  |  | 2.4*(1.6,3.3) |
| Venezuela | 1997-2016 | −1.3*(−2.3,−0.3) |  |  |  |  |  |  | −1.3*(−2.3,−0.3) |

***: p < 0.05 indicates statistical significance. APC: Annual Percent Change; AAPC: Average Annual Percent Change.**
