# Supplementary material for: Differences by sex and type of hypertension in mortality from hypertensive diseases between 1997 and 2020, and predictions for 2035 in Latin American and Caribbean countries
Source: PLoS One. 2026 Mar 2;21(3):e0342267. doi: 10.1371/journal.pone.0342267 (PMC12952635; doi:10.1371/journal.pone.0342267)
Supplement: S5 Table — (DOCX) [file pone.0342267.s008.docx]

**S5 Table. Average annual percent change and 95% confidence intervals for hypertension-mediated organ damage (I11-I13) for men in twenty countries in Latin America and the Caribbean, 1997 to 2020.**

| **Countries** | **Years** | **APC** | **Years** | **APC** | **Years** | **APC** | **Years** | **APC** | **AAPC** |
| --- | --- | --- | --- | --- | --- | --- | --- | --- | --- |
| Argentina | 1997−2013 | 0.7(−0.0,1.5) | 2013−2016 | 10.2(−6.5,29.9) | 2016−2020 | −4.1(−8.5,0.5) |  |  | 1.1(−1.1,3.3) |
| Brasil | 1997−2009 | 3.0*(2.5,3.5) | 2009−2015 | −3.4*(−4.8,−2.0) | 2015−2018 | 3.0(−3.0,9.4) | 2018−2020 | −4.1(−9.5,1.6) | 0.7(−0.3,1.6) |
| Chile | 1997−2005 | 1.6(−1.0,4.3) | 2005−2011 | −7.0*(−11.1,−2.7) | 2011−2014 | 11.0(−7.3,33.1) | 2014−2020 | −3.8*(−6.5,−1.2) | −1.0(−3.5,1.6) |
| Colombia | 1997−2001 | 5.1*(0.6,9.9) | 2001−2012 | −5.1*(−6.1,−4.1) | 2012−2015 | 5.2(−6.6,18.5) | 2015−2020 | −1.2(−3.5,1.1) | −1.2(−2.9,0.5) |
| Costa Rica | 1997−2006 | 4.3*(1.1,7.6) | 2006−2017 | −5.4*(−7.5,−3.2) | 2017−2020 | 13.8*(0.3,29.2) |  |  | 0.7(−1.5,2.9) |
| Cuba | 2001−2019 | 4.8*(4.2,5.4) |  |  |  |  |  |  | 4.8*(4.2,5.4) |
| Dominican Republic | 1999−2014 | 0.7(−0.4,1.9) | 2014−2020 | −14.1*(−22.4,−5.0) |  |  |  |  | −2.3*(−4.2,−0.3) |
| Ecuador | 1997−2012 | 10.6*(7.2,14.0) | 2012−2020 | −5.2*(−9.4,−0.8) |  |  |  |  | 4.8*(2.3,7.3) |
| El Salvador |  |  |  |  |  |  |  |  | NA |
| Guatemala | 2006−2020 | 6.6*(0.7,12.9) |  |  |  |  |  |  | 6.6*(0.7,12.9) |
| México | 1998−2021 | 2.8*(2.2,3.4) |  |  |  |  |  |  | 2.8*(2.2,3.4) |
| Nicaragua | 1997-2018 | 0.6(−0.2,1.4) | 2018-2020 | 36.8*(12.7,66.1) |  |  |  |  | 3.3*(1.5,5.1) |
| Panama | 1998-2012 | −1.7(−3.9,−1.2) | 2012-2016 | −23.5*(4.9,45.5) | 2016-2019 | 2.2(−9.5,15.6) |  |  | 3.2(−0.3,6.9) |
| Paraguay | 1997−2020 | 2.2*(1.3,3.0) |  |  |  |  |  |  | 2.2*(1.3,3.0) |
| Peru | 1999−2011 | −7.3*(−12.8,−1.5) | 2011−2014 | 65.0(−25.3,264.3) | 2014−2020 | 10.2*(3.2,17.5) |  |  | 5.7(−5.2,18.0) |
| Puerto Rico | 1999−2007 | −4.1*(−7.5,−0.6) | 2007−2012 | 10.4*(0.6,21.2) | 2012−2020 | −7.7*(−13.2,−1.9) |  |  | −1.3(−4.3,1.8) |
| Surinam | 1997−2014 | −4.3*(−7.1,−1.5) |  |  |  |  |  |  | −4.3*(−7.1,−1.5) |
| Trinidad and Tobago | 1999−2012 | −3.4*(−4.6,−2.1) |  |  |  |  |  |  | −3.4*(−4.6,−2.1) |
| Uruguay | 1997-2012 | 0.7(−0.2,1.6) | 2012-2018 | 7.4*(3.2,11.7) | 2018-2020 | −13.3(−26.6,2.3) |  |  | 1.0(−0.6,2.8) |
| Venezuela | 1997−2004 | 4.1*(0.7,7.6) | 2004−2009 | −4.3(−10.7,2.6) | 2009−2016 | 5.1*(2.5,7.8) |  |  | 2.2*(0.0,4.4) |

***: p < 0.05 indicates statistical significance. APC: Annual Percent Change; AAPC: Average Annual Percent Change, NA: Not applicable**
