# Supplementary material for: Differences by sex and type of hypertension in mortality from hypertensive diseases between 1997 and 2020, and predictions for 2035 in Latin American and Caribbean countries
Source: PLoS One. 2026 Mar 2;21(3):e0342267. doi: 10.1371/journal.pone.0342267 (PMC12952635; doi:10.1371/journal.pone.0342267)
Supplement: S6 Table — (DOCX) [file pone.0342267.s009.docx]

**S6 Table. Average annual percent change and 95% confidence intervals for hypertension-mediated organ damage (I11-I13) for women in twenty countries in Latin America and the Caribbean, 1997 to 2020.**

| **Countries** | **Years** | **APC** | **Years** | **APC** | **Years** | **APC** | **Years** | **APC** | **AAPC** |
| --- | --- | --- | --- | --- | --- | --- | --- | --- | --- |
| Argentina | 1997−2013 | 1.1*(0.3,1.9) | 2013−2016 | 11.1(−5.6,30.6) | 2016−2020 | −4.6*(−9.0,−0.1) |  |  | 1.3(−0.9,3.5) |
| Brasil | 1997−2009 | 0.9*(0.5,1.3) | 2009−2015 | −3.6*(−4.7,−2.5) | 2015−2018 | 1.7(−3.2,6.8) | 2018−2020 | −4.9*(−9.4, −0.3) | −0.7(−1.5,0.1) |
| Chile | 1997−2005 | 1.9(−0.7,4.5) | 2005−2011 | −6.9*(−11.1,−2.5) | 2011−2014 | 10.8(−9.1,35.0) | 2014−2020 | −3.8*(−6.6,−0.9) | −0.9(−3.6,1.9) |
| Colombia | 1997−1999 | 14.7(−1.5,33.7) | 1999−2012 | −5.2*(−6.0,−4.4) | 2012−2015 | 4.7(−8.1,19.4) | 2015−2020 | −2.1(−4.6,0.5) | −1.7(−3.7,0.4) |
| Costa Rica | 1997−2010 | 1.1(−0.5,2.7) | 2010−2016 | −9.7*(−15.0,−4.0) | 2016−2020 | 6.9(−1.5,16.1) |  |  | −0.9(−3.0,1.3) |
| Cuba | 2001−2019 | 4.4*(3.7,5.0) |  |  |  |  |  |  | 4.4*(3.7,5.0) |
| Dominican Republic | 1999−2014 | 0.6(−0.5,1.7) | 2014−2020 | −16.5*(−24.2,−7.9) |  |  |  |  | −2.9*(−4.8,−1.1) |
| Ecuador | 1997−2012 | 9.1*(6.6,11.6) | 2012−2020 | −4.6*(−8.0,−1.0) |  |  |  |  | 4.1*(2.2,6.1) |
| El Salvador |  |  |  |  |  |  |  |  | NA |
| Guatemala | 2005−2010 | 151.1*(67.6,276.1) | 2010−2020 | 2.9(−0.1,5.9) |  |  |  |  | 38.5*(22.7,56.4) |
| México | 1998−2020 | 1.0*(0.6,1.5) |  |  |  |  |  |  | 1.0*(0.6,1.5) |
| Nicaragua | 1997-2018 | 0.0(−1.0,1.2) | 2018-2020 | 24.0(−7.4,66.2) |  |  |  |  | 1.9(−0.6,4.6) |
| Panama | 1998-2012 | −3.0*(−5.4,−1.8) | 2012-2015 | −29.9*(−7.0,81.5) | 2015-2019 | 0.5(−6.5,8.2) |  |  | 1.3(−3.2,6.2) |
| Paraguay | 1997−2003 | −5.1(−10.8,0.9) | 2003−2006 | 16.2(−16.1,60.8) | 2006−2020 | 0.4(−0.8,1.6) |  |  | 0.8(−3.4,5.2) |
| Peru | 1999−2011 | −6.3*(−9.8,−2.6) | 2011−2015 | 43.7*(12.5,83.5) | 2015−2020 | 8.9*(2.6,15.6) |  |  | 5.4*(0.3,10.6) |
| Puerto Rico | 1999−2008 | −2.6*(−5.0,−0.2) | 2008−2011 | 12.7(−13.0,46.0) | 2011−2017 | −4.1*(−7.7,−0.4) |  |  | −0.7(−4.8,3.5) |
| Surinam | 1997−2014 | −5.2*(−7.0,−3.5) |  |  |  |  |  |  | −5.2*(−7.0,−3.5) |
| Trinidad and Tobago | 1999−2012 | −5.3*(−6.6,−4.0) |  |  |  |  |  |  | −5.3*(−6.6,−4.0) |
| Uruguay | 1997-2013 | −0.4(−1.4,0.6) | 2013-2017 | 11.1(−1.5,15.4) | 2017-2020 | −8.8(−18.7,2.19) |  |  | 0.3(−2.1,2.8) |
| Venezuela | 1997−2020 | −0.5(−1.3,0.3) |  |  |  |  |  |  | −0.5(−1.3,0.3) |

***: p < 0.05 indicates statistical significance. APC: Annual Percent Change; AAPC: Average Annual Percent Change, NA: Not applicable**
