# Supplementary material for: Differences by sex and type of hypertension in mortality from hypertensive diseases between 1997 and 2020, and predictions for 2035 in Latin American and Caribbean countries
Source: PLoS One. 2026 Mar 2;21(3):e0342267. doi: 10.1371/journal.pone.0342267 (PMC12952635; doi:10.1371/journal.pone.0342267)
Supplement: S7 Table — (DOCX) [file pone.0342267.s010.docx]

**S7 Table.** Number of primary hypertension (I10) deaths, age-standardized mortality rates, and percentage change in cases due to population growth and risk among men in Latin America and the Caribbean, 2020 and predicted 2035.

| Countries | Male population (annual million) | | Number of deaths in men | | Age-standardized mortality rates | | Change total (%) | Change due to population (%) | Change due to risk (%) |
| --- | --- | --- | --- | --- | --- | --- | --- | --- | --- |
|  | 2020 | 2035 | 2020 | 2035 | 2020 | 2035 |  |  |  |
| Argentina | 22.0 | 24.9 | 6080 | 7135 | 3.9 | 3.3 | 17.4 | 42.2 | -24.9 |
| Brazil | 103.3 | 111.1 | 65294 | 151539 | 15.8 | 12.9 | 132.1 | 124.0 | 8.1 |
| Chile | 9.3 | 9.8 | 5146 | 14042 | 7.8 | 9.2 | 172.9 | 89.8 | 83.1 |
| Colombia | 24.3 | 26.8 | 7446 | 19492 | 8.6 | 6.1 | 161.8 | 163.7 | -1.9 |
| Costa Rica | 2.5 | 2.8 | 444 | 2392 | 4.5 | 5.8 | 438.8 | 153.1 | 285.8 |
| Cuba | 5.6 | 5.4 | 2226 | 7238 | 4.1 | 8.6 | 225.2 | 49.0 | 176.2 |
| Dominican Republic | 5.4 | 6.0 | 7102 | 32882 | 25.0 | 70.4 | 363.0 | 65.6 | 297.5 |
| Ecuador | 8.5 | 10.4 | 3203 | 2455 | 14.4 | 2.5 | -23.3 | 121.7 | -145.0 |
| Guatemala | 8.3 | 11.3 | 1856 | 4381 | 9.6 | 8.0 | 136.1 | 92.4 | 43.7 |
| Mexico | 60.6 | 71.2 | 16217 | 27330 | 7.0 | 4.8 | 68.5 | 62.6 | 6.0 |
| Nicaragua | 3.2 | 3.8 | 365 | 213 | 4.5 | 1.0 | -41.4 | 152.9 | -194.3 |
| Panama | 20.5 | 26.0 | 874 | 1594 | 8.4 | 5.6 | 82.4 | 130.3 | -47.8 |
| Paraguay | 3.2 | 4.2 | 1659 | 5448 | 16.1 | 19.4 | 228.4 | 110.4 | 118.0 |
| Peru | 15.9 | 18.5 | 1978 | 93 | 7.0 | 0.1 | -95.3 | 89.5 | -184.9 |
| Puerto Rico | 1.6 | 1.3 | 1004 | 1113 | 4.6 | 4.3 | 10.9 | 30.1 | -19.1 |
| Uruguay | 1.7 | 1.8 | 630 | 977 | 3.8 | 4.7 | 55.1 | 34.2 | 20.9 |
| Venezuela | 15.2 | 17.1 | 1808 | 7 | 6.3 | 0.0 | -99.6 | 102.6 | -202.2 |
