# Supplementary material for: Differences by sex and type of hypertension in mortality from hypertensive diseases between 1997 and 2020, and predictions for 2035 in Latin American and Caribbean countries
Source: PLoS One. 2026 Mar 2;21(3):e0342267. doi: 10.1371/journal.pone.0342267 (PMC12952635; doi:10.1371/journal.pone.0342267)
Supplement: S8 Table — (DOCX) [file pone.0342267.s011.docx]

**S8 Table.** Number of primary hypertension (I10) deaths, age-standardized mortality rates, and percentage change in cases due to population growth and risk among women in Latin America and the Caribbean, 2020 and predicted 2035.

| Countries | Female population (annual million) | | Number of deaths in men | | Age-standardized mortality rates | | Change total (%) | Change due to population (%) | Change due to risk (%) |
| --- | --- | --- | --- | --- | --- | --- | --- | --- | --- |
|  | 2020 | 2035 | 2020 | 2035 | 2020 | 2035 |  |  |  |
| Argentina | 22.4 | 25.9 | 7491 | 8772 | 2.5 | 2.2 | 17.1 | 33.6 | -16.5 |
| Brazil | 106.7 | 116.1 | 75326 | 166156 | 12.0 | 9.0 | 120.6 | 138.4 | -17.8 |
| Chile | 9.4 | 10 | 8066 | 19441 | 6.8 | 8.3 | 141.0 | 70.3 | 70.7 |
| Colombia | 24.9 | 27.8 | 8670 | 18194 | 6.6 | 4.1 | 109.9 | 159.2 | -49.4 |
| Costa Rica | 2.5 | 2.8 | 521 | 3078 | 3.6 | 4.8 | 490.9 | 148.6 | 342.4 |
| Cuba | 5.6 | 5.5 | 2224 | 7611 | 2.8 | 6.5 | 242.3 | 59.3 | 183.0 |
| Dominican Republic | 5.4 | 6 | 6797 | 339663 | 27.2 | 52.3 | 399.7 | 75.9 | 323.8 |
| Ecuador | 8.5 | 10.4 | 3338 | 2568 | 9.5 | 1.9 | -23.1 | 112.6 | -135.7 |
| Guatemala | 8.5 | 11.5 | 2251 | 5815 | 7.1 | 8.0 | 158.3 | 105.7 | 52.7 |
| Mexico | 63.2 | 74.5 | 20556 | 28449 | 5.7 | 3.5 | 38.4 | 75.6 | -37.2 |
| Nicaragua | 3.3 | 4 | 432 | 236 | 2.1 | 0.7 | -45.3 | 163.9 | -209.2 |
| Panama | 2.1 | 2.6 | 859 | 1600 | 5.6 | 4.0 | 86.3 | 134.0 | -47.7 |
| Paraguay | 3.2 | 4.1 | 1695 | 4147 | 11.8 | 11.3 | 144.7 | 99.7 | 45.0 |
| Peru | 16.2 | 18.7 | 2082 | 130 | 6.3 | 0.1 | -93.7 | 109.1 | -202.8 |
| Puerto Rico | 1.8 | 1.5 | 1214 | 1782 | 3.6 | 3.0 | 46.9 | 59.2 | -12.3 |
| Uruguay | 1.7 | 1.8 | 955 | 1211 | 2.9 | 3.1 | 26.8 | 20.5 | 6.3 |
| Venezuela | 15.4 | 17.7 | 3810 | 12988 | 4.5 | 7.2 | 240.0 | 131.0 | 109.9 |
