# Supplementary material for: Differences by sex and type of hypertension in mortality from hypertensive diseases between 1997 and 2020, and predictions for 2035 in Latin American and Caribbean countries
Source: PLoS One. 2026 Mar 2;21(3):e0342267. doi: 10.1371/journal.pone.0342267 (PMC12952635; doi:10.1371/journal.pone.0342267)
Supplement: S9 Table — (DOCX) [file pone.0342267.s012.docx]

**S9 Table.** Number of hypertension-mediated organ damage (HMOD) (I11-I13) deaths, age-standardized mortality rates, and percentage change in cases due to population growth and risk among men in Latin America and the Caribbean, 2020 and predicted 2035.

| Countries | Male population (annual million) | | Number of deaths in men | | Age-standardized mortality rates | | Change total (%) | Change due to population (%) | Change due to risk (%) |
| --- | --- | --- | --- | --- | --- | --- | --- | --- | --- |
|  | 2020 | 2035 | 2020 | 2035 | 2020 | 2035 |  |  |  |
| Argentina | 22 | 24.9 | 10907 | 19132 | 6.7 | 8.3 | 75.4 | 45.0 | 30.4 |
| Brazil | 103.3 | 111.1 | 64145 | 134757 | 11.8 | 11.3 | 110.1 | 126.2 | -16.1 |
| Chile | 9.3 | 9.8 | 7697 | 13167 | 9.6 | 8.9 | 71.1 | 85.0 | -13.9 |
| Colombia | 24.3 | 26.8 | 15403 | 39019 | 13.4 | 13.2 | 153.3 | 163.3 | -10.0 |
| Costa Rica | 2.5 | 2.8 | 1774 | 4294 | 12.3 | 11.3 | 142.1 | 137.2 | 4.8 |
| Cuba | 5.6 | 5.4 | 9788 | 24041 | 18.6 | 26.7 | 145.6 | 51.4 | 94.2 |
| Dominican Republic | 5.4 | 6 | 1587 | 1103 | 5.1 | 2.3 | -30.5 | 66.7 | -97.2 |
| Ecuador | 8.5 | 10.4 | 5943 | 7322 | 16.3 | 7.3 | 23.2 | 119.9 | -96.7 |
| Guatemala | 8.3 | 11.3 | 1382 | 2630 | 6.3 | 4.8 | 90.4 | 89.5 | 0.9 |
| Mexico | 60.6 | 71.2 | 41809 | 88399 | 15.6 | 16.5 | 111.4 | 60.1 | 51.3 |
| Nicaragua | 3.2 | 3.8 | 2576 | 11498 | 42.6 | 52.4 | 346.4 | 129.8 | 216.6 |
| Panama | 20.5 | 26 | 1453 | 8753 | 12.8 | 35.9 | 502.5 | 121.3 | 381.2 |
| Paraguay | 3.2 | 4.2 | 2043 | 4598 | 14.6 | 16.3 | 125.1 | 112.1 | 13.0 |
| Peru | 15.9 | 18.5 | 2924 | 11283 | 4.6 | 6.2 | 285.9 | 94.3 | 191.6 |
| Puerto Rico | 1.6 | 1.3 | 1004 | 1113 | 10.3 | 4.3 | 10.9 | 30.1 | -19.1 |
| Uruguay | 1.7 | 1.8 | 1221 | 2391 | 6.1 | 10.7 | 95.9 | 36.2 | 59.7 |
| Venezuela | 15.2 | 17.1 | 16600 | 44370 | 29.0 | 36.4 | 167.3 | 113.3 | 54.0 |
