# Supplementary material for: Differences by sex and type of hypertension in mortality from hypertensive diseases between 1997 and 2020, and predictions for 2035 in Latin American and Caribbean countries
Source: PLoS One. 2026 Mar 2;21(3):e0342267. doi: 10.1371/journal.pone.0342267 (PMC12952635; doi:10.1371/journal.pone.0342267)
Supplement: S10 Table — (DOCX) [file pone.0342267.s013.docx]

**S10 Table.** Number of hypertension-mediated organ damage (HMOD) (I11-I13) deaths, age-standardized mortality rates, and percentage change in cases due to population growth and risk among women in Latin America and the Caribbean, 2020 and predicted 2035.

| Countries | Female population (annual million) | | Number of deaths in men | | Age-standardized mortality rates | | Change total (%) | Change due to population (%) | Change due to risk (%) |
| --- | --- | --- | --- | --- | --- | --- | --- | --- | --- |
|  | 2020 | 2035 | 2020 | 2035 | 2020 | 2035 |  |  |  |
| Argentina | 22.4 | 25.9 | 14874 | 25153 | 4.4 | 6.0 | 69.1 | 33.6 | 35.5 |
| Brazil | 106.7 | 116.1 | 69513 | 14150 | 8.5 | 7.8 | 103.6 | 134.4 | -30.8 |
| Chile | 9.4 | 10 | 10298 | 16223 | 8.2 | 7.3 | 57.5 | 70.0 | -12.5 |
| Colombia | 24.9 | 27.8 | 15661 | 35648 | 9.1 | 8.9 | 127.6 | 153.3 | -25.6 |
| Costa Rica | 2.5 | 2.8 | 1703 | 2915 | 7.8 | 5.5 | 71.2 | 137.0 | -65.9 |
| Cuba | 5.6 | 5.5 | 9472 | 22532 | 13.7 | 18.4 | 137.9 | 59.0 | 78.9 |
| Dominican Republic | 5.4 | 6 | 1363 | 786 | 5.6 | 1.2 | -42.3 | 77.3 | -119.6 |
| Ecuador | 8.5 | 10.4 | 6142 | 8188 | 10.9 | 6.3 | 33.3 | 109.7 | -76.4 |
| Guatemala | 8.5 | 11.5 | 1508 | 3063 | 4.8 | 4.1 | 103.1 | 103.4 | -0.3 |
| Mexico | 63.2 | 74.5 | 47276 | 87393 | 13.0 | 11.8 | 84.9 | 74.1 | 10.8 |
| Nicaragua | 3.3 | 4 | 1977 | 6821 | 20.9 | 20.2 | 245.0 | 154.3 | 90.7 |
| Panama | 2.1 | 2.6 | 1188 | 4912 | 7.2 | 14.7 | 313.5 | 127.1 | 186.4 |
| Paraguay | 3.2 | 4.1 | 1989 | 4178 | 10.8 | 11.9 | 110.1 | 99.9 | 10.2 |
| Peru | 16.2 | 18.7 | 3193 | 17407 | 3.5 | 7.5 | 445.2 | 109.5 | 335.6 |
| Puerto Rico | 1.8 | 1.5 | 2281 | 2709 | 6.4 | 4.3 | 18.8 | 56.2 | -37.4 |
| Uruguay | 1.7 | 1.8 | 1758 | 3037 | 4.1 | 7.7 | 72.8 | 20.1 | 52.6 |
| Venezuela | 15.4 | 17.7 | 4918 | 996 | 15.5 | 0.5 | -79.7 | 129.8 | -209.6 |
