# Supplementary material for: Differences by sex and type of hypertension in mortality from hypertensive diseases between 1997 and 2020, and predictions for 2035 in Latin American and Caribbean countries
Source: PLoS One. 2026 Mar 2;21(3):e0342267. doi: 10.1371/journal.pone.0342267 (PMC12952635; doi:10.1371/journal.pone.0342267)
Supplement: Attachment — Submitted filename: 5 Comments.docx [file pone.0342267.s015.docx]

Unify the typing of “Peru” and “Perú” in the affiliations

Keywords: Please use all in English

Introduction, paragraph 2, line 4: “(LAC), 2019, the prevalence ” connector seems to be missing

Introduction, paragraph 2, line 7: “12),as well” space is needed

Introduction, paragraph 3, line 1: “growth of the elderly population, have led to a growing” can improve writting

Introduction, paragraph 3, line 3: “(16).These “ space is needed

Materials and methods, paragraphs 2, line 2: “2020 (or the most recent available year)” please clarify that

Materials and methods, paragraphs 2, line 9: “Mortality data were analyzed in five-year age intervals (0–4, 5–9, 10–14, ..., 85+ years).” this is not shown in the results

Materials and methods, Predictions through 2035, line 11: “disease risk” It would be great to elaborate more on how you estimated variation in disease risk

Results, general comments: The text uses some name in spanish, needs translation (ej: Dominican Republic). Also, for figures, the resolution is subotpimal to confirm the numbers are accurate.

Results, paragraph 2, line 4: “ Trinidad and Tobago (AAPC: −5.7%) showed a significant decreasing trend” why is that? This is theoretically unexpected, and no mention of this finding is found in results. Results, paragraph 2, line 7: “ Republica Dominicana” need to unify the use of the name in the text and in the graph

Results, paragraph 3, line 1: “ mortality trends” it would be great to show the graph trends

Results, paragraph 3, Figures and supplements: the figure does not seem to represent what is stated in the text, and the supplements are not available for review

Results, paragraph 4, line 5: “This sequence -if not largely influenced by changes in certification- suggests a significant structural shift in the disease’s trajectory, potentially influenced by changes in access to treatment, health policy adjustments, or broader socioeconomic factors.” and line 11 “These fluctuations underscores the instability of mortality trends and highlight in the country and the need for sustained public health interventions” : this is part of the discussion, not the results

Results, paragraph 4, lines 13 and 22: the figure does not seem to represent what is stated in the text, and the supplements are not available for review

Results, last paragraph, line 1: “HTA” usually in english hypertension is abbreviated as HTN, and also need to show where was used abbreviation before (unable to find)

Results, last paragraph, line 2: “risk” how was it defined?

Results, last paragraph, line 4: “saw a decrease” and all the “saw” use: since this is a projection, saw is a stated past writting. Suggest to use something future-trend like “is estimated that…. could… might”, would be more accurate.

Results, last paragraph, line 6: “ stronger increase in risk factors” which risk factors?, how did you estimated them and how did you estimated the expected change on them?

Discussion, paragraph 8 (In Peru, the…), line 10: “(refs)” and line 15 “(Ref. Pizzato et al. 2024)”: Improve structure in references

References: add the date of consult for webpages as they tend to change.

Table 1, title: “ cancer deaths”: why cancer deaths?

Table 1, categories: “Relative change in”: improve structure as it is confusing, and if abbreviations are used needs an explanation at the bottom of the table

Table 1, “Republica Dominicana” unify the name of the country in all the manuscript

Table 2, title: “ cancer deaths”: why cancer deaths?

Table 2, “Number of deaths in men”: I understand this table is for women, please adjust or clarify

Table 2, categories: “Relative change in”: improve structure as it is confusing, and if abbreviations are used needs an explanation at the bottom of the table

Table 2, “Republica Dominicana” unify the name of the country in all the manuscript
