# Supplementary material for: Differences by sex and type of hypertension in mortality from hypertensive diseases between 1997 and 2020, and predictions for 2035 in Latin American and Caribbean countries
Source: PLoS One. 2026 Mar 2;21(3):e0342267. doi: 10.1371/journal.pone.0342267 (PMC12952635; doi:10.1371/journal.pone.0342267)
Supplement: Attachment — Submitted filename: Response to Reviewers.docx [file pone.0342267.s019.docx]

16 January, 2026

Emily Chenette

Senior Editor of Plos One

Reference: PONE-D-25-57467R1

Dear Dra. Emily Chenette

We greatly appreciate the consideration given to our manuscript and the valuable comments made by the reviewers. Below we present a point-by-point response to the comments. As requested, we have highlighted the changes that were made from the previous version in the attached revised document.

We are grateful for the opportunity to publish our research work in your new journal. All co-authors have seen and agree with this resubmission.

Thank you for your consideration of our manuscript.

Sincerely,

J. Smith Torres Román, MD

Universidad Científica del Sur, Lima, Peru

Tel: +(51) 99 321-9508

Email: [jstorresroman@gmail.com](mailto:jstorresroman@gmail.com)

**Reviewer #1: Thanks for addressing the methodologic concerns.**

Introduction, line 4, leave an space “[3].Though”.

Authors: Done

Put a dot at the end of 2nd paragraph of the introduction and also at the 2nd paragraph of materials and methods.

Authors: Done

Predictions through 2035, last line, leave a space “trends[25].”

Authors: Done

Results: start by text and at the end put “figure 1” at the end of the 1st line.

Authors: Done, although it would no longer be necessary to place Figure 1 at the end of the text, since each sentence has the figure, number, and letter to which it belongs.

Resuls, Trends for primary hypertension, “(3.4%),Chile” needs an space, “(4.7%) El Salvador (7.7%), “ a comma is missing.

Authors: Done

Make sure graph quality is optimal for publication.

Authors: Done

**Reviewer #2: All comments have been addressed**

Authtros should be complimented because they evaluated and answered appropriately to all our comments. The work is ready now to be published and improved.

Authors: We greatly appreciate the valuable comments that have significantly improved our study.
